# Beyond clinical trials: Extending the role of the clinical research nurse into social care and homeless research

**DOI:** 10.1111/jocn.15911

**Published:** 2021-06-24

**Authors:** Elizabeth Biswell R, Michael Clark, Michela Tinelli, Gillian Manthorpe, Joanne Neale, Martin Whiteford, Michelle Cornes

**Affiliations:** ^1^ Health and Social Care Workforce Research Unit King's College London London UK; ^2^ Care Policy and Evaluation Centre The London School of Economics and Political Science London UK; ^3^ National Addiction Centre Institute of Psychiatry, Psychology & Neuroscience King's College London London UK; ^4^ Department of Nursing & Community Health Glasgow Caledonian University Glasgow UK

**Keywords:** caring‐recruiting dichotomy, clinical research nurse, hard to reach, homelessness, social care research, study recruitment, workforce development

## Abstract

**Aim:**

Clinical research nurses work at the fulcrum of clinical trials with clearly defined roles and responsibilities. In England, the National Institute for Health Research (the main funder of health research) has broadened its scope to encompass social care research. The expectation is that clinical research nurses will expand their skill set to support these new studies, many of which will employ qualitative and mixed methods. This discussion paper explores the challenges of facilitating this clinical academic workforce development through a case study of a homeless health and social care research project. This was one of the first studies to engage clinical research nurses in this new and expanded role.

**Background:**

Much of what is known about the research nurse workforce has been generated through studies of clinical trials in oncology. The *‘caring*‐*recruiting’* dichotomy has been used as a heuristic device for identifying workforce issues that can impact on study delivery such as how intense pressure to recruit study participants leads to low job satisfaction.

**Design:**

This case study reflects on the authors' experiences of employing a clinical research nurse in a social care research project concerned with the discharge of homeless people from hospital. The *‘caring*‐*recruiting’* dichotomy is used to generate new information about the relationship between workforce development and the successful delivery of social care research.

**Conclusion:**

The case study illuminates how social care research can generate different pressures and ethical challenges for research nurses. The time and skill it took to recruit study participants identified as *‘hard to reach’* was suggestive of the need to move beyond performance measures that prioritise recruitment metrics. The need for different types of staff supervision and training was also warranted as supporting study participants who were homeless was often distressing, leading to professional boundary issues.

**Relevance to workforce development:**

This study highlights that performance management, training and supervisory arrangements must be tailored to the characteristics of each new study coming onto the portfolio to ensure research nurses are fully supported in this new and expanded role.

## 1 AIMS

In England, the National Institute for Health Research (NIHR) has recently widened its focus to provide more support for social care research that aims to improve quality of life for users and carers through better social care provision and practice (NIHR, 2020). To support this development, the expectation is that clinical research nurses working mainly in clinical trials across 15 Clinical Research Networks (CRNs) will become increasingly involved in supporting recruitment to social care research. This is likely to take the work of clinical research nurses into settings beyond those in which many have so far worked, perhaps working with new methodological approaches and discussing topics with client groups with whom they are less familiar.

This discussion paper reflects on the challenges of facilitating this workforce development by drawing on the learning from a health and social care study about improving hospital discharge arrangements for people who are homeless (Cornes et al., 2019; Cornes et al., forthcoming). We describe how clinical research nurses were engaged in the delivery of this mixed methods, longitudinal study spanning NHS and non‐NHS settings. Recruitment to the study was particularly challenging as many of the study participants could be identified as *‘hard to reach’*. This reflects that most of the prospective participants were experiencing extreme forms of social exclusion (e.g. where there is overlap between sleeping rough on the streets, substance misuse, mental health problems and poor physical health). We reflect on the challenges encountered by the research nurse in day‐to‐day study delivery and describe how they were overcome. We explore the implications of these experiences for staff supervision, training and performance management. Finally, we consider the advantages for social care research that can flow from the deployment of clinical research nurses as uniquely skilled care and research professionals.

## 2 BACKGROUND

Clinical research nurses are registered nurses who are usually employed within research sites to help facilitate and conduct any phase of a clinical trial (Gibbs & Lowton, 2012). Many research nurses will refer to themselves as ‘clinical nurse specialists’. The NIHR in England and equivalent structures in Northern Ireland, Wales and Scotland have funded a significant expansion in the clinical research nurse workforce. Reliable data on the number and activity of clinical research nurses in the United Kingdom are difficult to obtain; however, approximately 15,000 research staff, of whom 10–12,000 are estimated to be clinical research nurses, currently assist in the delivery of clinical trials (Tinkler et al., 2018).

The clinical role of nurses in research has been most thoroughly documented and described in oncology clinical trials (Bevans et al., 2011). However, there has been less clarity about the role of nurses in research studies outside of the oncology speciality. In a qualitative study, examining the perspectives of 11 CRNs in the United Kingdom, Kunhunny and Salmon (2017) report how this lack of clarity along with limited professional development opportunities works to undermine the CRN identity and optimism about progression in the future.

According to Pick et al. (2010), a solid foundation based on years of experience in nursing is vital to the clinical research nurse role which may include recruitment, taking consent to study participation, collecting data from patient records, follow up and other tasks. While non‐clinical researchers would maintain clear boundaries between research and care, there is some overlap in the clinical research nurse role. The International Association of Clinical Research Nurses (IACRN) defines clinical research nursing as: ‘The specialized practice of professional nursing focused on maintaining equilibrium between care of the research participant and fidelity to the research protocol'. This specialty practice incorporates human subject protection; care coordination and continuity; contribution to clinical science; clinical practice and study management throughout a variety of professional roles, practice settings and clinical specialties (IACRN, 2016). More recently, the NIHR has worked with the Royal College of Nursing (RCN) to develop clinical research nurse competencies and career pathways (NIHR, 2017)

Despite investment in clinical research nurses and their infrastructure, the successful recruitment of sufficient patients into research studies remains challenging. A growing body of literature is exploring barriers to successful study delivery and is also seeking to understand more about the reality of practising in the clinical research nurse role (Camsooksai et al., 2013). Tinkler et al. (2018) undertook a qualitative study exploring the experiences of 19 clinical research nurses with an emphasis on factors that may impact on successful study delivery. They draw attention to the emergence of a potential ‘*caring*‐*recruiting’* dichotomy, whereby clinical research nurses may, in some circumstances, experience tension in their double responsibilities of, on the one hand, caring for their patients and, on the other, meeting the expectations with regard to recruitment targets. They describe how clinical research nurses can feel pressurised by, for example, medical doctors who push for recruitment at all costs, and the ethical issues that arise when they feel the patient may not truly understand the implications of taking part. The nurses described feeling especially pressured when working on industry‐funded (rather than academic) studies where recruitment targets were high. They described feeling like reluctant salespeople, whether interacting in the organisation to gain support for the delivery of a study or approaching patients regarding study participation. Tinkler et al. (2018) concluded that balancing the experience of the ‘*caring*‐*recruiting’* dichotomy could reduce recruitment problems and increase successful delivery of a range of research projects.

## 3 DESIGN

This discussion paper is based on the authors' experiences of undertaking research social care in the field of homelessness. The homeless hospital discharge study was commissioned by the National Institute of Health Research (NIHR) (2015–2019) and sought to provide a longitudinal understanding of the housing and social care trajectories of patients leaving hospital, and following them up in the community for a period of 3 months. As this social care study was one of the first to employ a clinical research nurse, it affords an opportunity to reflect on the workforce developments described above. We begin by providing a brief overview of the homeless research and its methods before considering the role of clinical research nurses therein. We use the *‘caring*‐*recruiting’* (Tinkler et al., 2018) dichotomy as a heuristic device for identifying workforce issues that can impact on study delivery. Ethical approval for the research was obtained from the London and South East Research Ethics Committee in April 2016 (16/EE/0018). The research is concordant with RAMESES II Equator Checklist.

### 3.1 Background to homeless hospital discharge study

Homelessness is a complex phenomenon that covers a wide range of circumstances including people sleeping rough, single homeless people living in hostels, shelters, temporary supported accommodation and staying with friends and family (‘sofa surfing’). In England, homelessness has increased by 165% since 2010 (Homeless Monitor, 2018). In this same period, attendance at emergency departments by people experiencing homelessness has trebled and there has also been a steep rise in hospital admissions (BMA, 2019).

In 2013, it was reported that 70% of homeless patients were discharged back to the street without having their housing, care and support needs addressed (DH, 2013). The research reported here evaluated a range of Homeless Hospital Discharge (HHD) schemes delivered by the voluntary sector in England to improve the discharge experiences of homeless patients. Schemes comprised the co‐location of housing workers in hospitals in order to help patients find accommodation as well as ‘step‐down’ facilities where patients could recover outside hospital while they found somewhere safe to stay.

### 3.2 ‘Hard to reach’

People who are homeless are often conceptualised as *‘hard to reach’*, meaning that it can be difficult for researchers to recruit them to studies and maintain contact (Sydor, 2013). This was acknowledged in the original NIHR commissioning brief for the hospital discharge study, where it was reported that the homeless population poses particular challenges for research in terms of participation and consent (NIHR, 2014).

In a systematic review of the literature on undertaking health research with socially disadvantaged groups, Bonevski et al. (2014) identify the main reasons for recruitment problems as being: mistrust in research or researchers; fear of authority; gatekeepers who restrict access to health research where they might have encouraged participation and perceptions that participation will present no personal or community benefit and may cause potential harm, stigma, mistreatment or exploitation. Successful strategies for overcoming these are identified as: community group–researcher partnerships; incentives and gifts; building relationships and trust; using peer researchers and using accessible language. All these strategies were employed to some degree in the study protocol for the hospital discharge study, but they did not overcome all the recruitment problems encountered.

## 4 METHODS USED IN THE MAIN STUDY

The homeless hospital discharge study was a realist evaluation comprising three inter‐linked work packages (WPs). The overall aim was to establish the effectiveness and cost‐effectiveness of specialist homeless hospital discharge arrangements (as compared to hospital sites with no specialist provision). Clinical research nurse involvement was limited to WP2, which was an economic evaluation. In WP2, the aim was to recruit 378 homeless patients (to achieve statistical significance) across four hospital sites. Patients were to be contacted at two time points (shortly after discharge and again 3 months later) and asked to complete a survey questionnaire. The questionnaire included a range of questions on health status (e.g. HIV status) and was designed to track recovery and a range of other outcomes (e.g. smoking cessation and drug use). The questionnaire was also designed to gather information on wider public service utilisation costs post‐discharge (e.g. use of social care, nights in prison, etc.). The survey also incorporated the EQ‐5D tool to measure quality of life and user satisfaction with the service received.

Across the four case study sites, it was planned that the survey questionnaire would be administered by staff employed in the HHD schemes as part of their routine case management (monitoring and review) process. This was considered feasible as the pilot site was already using a similar questionnaire (monitoring) pro forma to generate evidence on outcomes for its commissioners. It was also planned that these community/researcher partnerships would be underpinned by a small financial incentive (£400 for each service plus £20 for every questionnaire completed).

In the original protocol, the research team did not plan for the inclusion of clinical research nurses because we were not aware that their role might extend beyond clinical trials. Their involvement came about opportunistically midway through the study when we needed help to rescue our failing WP2 recruitment strategy.

## 5 RECRUITMENT CHALLENGES

Recruiting in hospitals and following up homeless patients for WP2 proved extremely challenging. As noted above, while we had planned that the HHD schemes would include the survey questionnaire as part of their routine monitoring and review, this proved unworkable across all but one of the sites. With the benefit of hindsight, the pilot HHD scheme with which we had initially worked had been somewhat unusual in having robust monitoring and review procedures in place. We later discovered that one of the HHD schemes did not undertake any form of monitoring and review of their patients once they left hospital. With the exception of one, all the HHDs reported that their staff did not have time to ask patients to complete questionnaires (which were perceived as overly long and burdensome). Another barrier was that the ‘housing workers’ in three of the HHD schemes reported feeling uncomfortable asking health questions, especially those linked to stigmatised conditions such as HIV.

The one site that did achieve its targets for completing the questionnaires employed a former clinical research nurse as the HHD scheme manager. She commented that this kind of work was entirely familiar to her and not a problem. She encouraged and motivated her team of workers to complete the requisite number of questionnaires to great effect. On her recommendation, we contacted the CRNs linked to the other research sites and were able to recruit a clinical research nurse for Site 1 (the hospital with the largest numbers of homeless patients in this study). Unfortunately, it was not possible to recruit a research nurse for the two other remaining sites as staff did not have the time to take on any additional work. Recruitment continued to be a significant challenge in these other two sites and while we collected 238 questionnaires at baseline and 176 at follow up (across all four HHD sites), this was disappointingly short of the overall target of 378 responses. The lack of primary questionnaire data meant that the economic evaluation had to adopt a different approach that departed from the original protocol (Cornes et al., forthcoming). These disappointing results reinforced a key learning point for the research team, namely about the importance of involving the CRNs and clinical research nurse colleagues at the earliest opportunity. Indeed, there is an urgent need for CRNs to raise awareness among the social care and homeless academic research community about the valuable role clinical research nurses can play in these areas to foster such engagement and partnerships working.

We now turn our attention to the day‐to‐day challenges faced by the clinical research nurse tasked with delivering the study in Site 1.

### 5.1 Identification of homeless patients on the ward

In Site 1, the clinical research nurse managed to recruit the target numbers of homeless patients in a short time. A major advantage here was that the research nurse had worked in the hospital for some years and was familiar with ward staff and the computer systems for identifying homeless patients. Identifying hospital patients who are homeless can be challenging as homelessness is not routinely recorded in hospital data. Often the post code ZZZ is used to signify that the patient may be homeless. Each morning the research nurse would look at new admissions for each ward, searching for this postcode and compiling a list of potential participants. Making enquiries about new admissions with ward staff (with whom she was already familiar) also enabled identification of other patients who may have been homeless but who did not have the ZZZ code listed.

Having a clinical research background was advantageous for recruiting homeless patients in hospital. Once a homeless patient was identified, the researcher took time to look through the admission notes to ascertain what had brought them into hospital. Understanding different presenting conditions was important in order to make decisions about if and when to approach a prospective participant. For example, those who had been admitted with acute psychosis, agitation or injury due to substance misuse would often be experiencing drug withdrawal. It would be important here to check that substitute prescribing had been arranged before broaching the topic of research. In addition to understanding the clinical significance of any agitated, anxious or aggressive behaviour, background information was also important so as not to cause the patient to have to repeat their story to yet another health professional and to help overcome suspicion by *‘knowing’* the patient. A clinical understanding of the circumstances of the patient could, then, put the person at ease and be a helpful foundation for establishing the kind of relationship needed to recruit someone to a study.

Nursing involves not only establishing a full medical and psychosocial history but also performing an observational assessment when approaching a patient's bed area. This often occurs as an automatic *‘head to toe’* scan of the patient and their surrounding area as the nurse approaches. This scan frequently led the research nurse to observe that the most basic needs of the patient had not been met; for example, the patient may have had unclean hair, face and hands; cuts that had not been cleaned (perhaps an indication of self‐harm) or clothes that were torn, dirty or wet (often a bag of these clothes would be left at the foot of the bed while the patient had been dressed temporarily in a hospital gown). That these basic needs are overlooked by other hospital staff may reflect the stigma and lack of understanding (cultural distance) that patients who are homeless often face when in hospital.

In addressing the unmet needs of homeless patients on the ward, the research nurse was aware that there was a second‐hand clothing cupboard located in the hospital and, with help from the chaplaincy and an outside charity, was able to source clothing supplies and toiletries. These could be offered to the patients to help them maintain their hygiene and have a clean change of clothes. A washing machine and tumble dryer were found on the older people care wards, which meant dirty clothes could be washed. This compassionate kindness on the part of the research nurse undoubtedly encouraged participation in the study as patients often expressed gratitude for this help. While there was potential for conflict with HHD staff given that it was part of their role to provide these basic necessities for homeless patients, overwhelming pressures on the service meant that they were generally grateful for the practical support being provided additionally to their patients by the research nurse.

### 5.2 Consent

In securing formal consent to the study, patients were informed that they were under no obligation to take part and those who declined would receive the same care and attention. Most patients were pleased to hear that this research was being conducted and wanted to take part as they said that they felt the topic was important. Additionally, each participant was given a £10 shopping voucher for taking part. As many homeless patients come into hospital without any cash (or means of accessing it), the extent to which this is appreciated should not be underestimated.

### 5.3 Administering the questionnaire at Time 1

While the housing workers had felt uncomfortable asking certain health questions, the research nurse was confident and used to asking these types of questions, having already worked on studies covering drug (mis)use and associated conditions. However, the research nurse did not always feel comfortable asking the survey questions concerned with public resource utilisation, such as the number of times the patient had been arrested or in prison. Patients were often suspicious of these questions and of their relevance to their health. The EQ‐5D scale also raised an interesting issue in that participants are asked to rate their health on a scale of 1–10. In answering this, participants often wanted to make the distinction between their mental and physical health, commenting that their scores would be very different for each. Many of the homeless patients taking part in the study were experiencing anxiety and depression.

Encounters with patients were often lengthy and extended far beyond the actual administration of the survey. Most had few visitors, so often wanted to talk in depth about their experiences of life and on the streets. Often men seemed to want to talk about their self‐harming, violence or criminal behaviour; while women wanted to talk about past relationships and their children who may have been placed in the care of the state. Box 1 provides an insight into the issues that the research nurse may face when seeking to recruit patients who are homeless. Again, a research nurse with a range of clinical and research experience is able to understand these issues and rapidly consider their impact on the person and on recruitment and data collection process for each individual.

BOX 1 **Admitted from the streets.**

Caleb (names have been changed) was bought into the emergency department (ED) late one Saturday night after collapsing and having a seizure in the street. On the night he was bought into hospital it had been snowing heavily and he had lain in the street for 20 min until an ambulance called by a passer‐by had arrived. He had experienced urinary incontinence during the seizure. A witness related his seizure to the use of Spice (a synthetic cannabinoid).

On Monday following handover of weekend ED admissions to the clinical research nurse, Caleb was awaiting discharge from the admissions ward when he was approached to take part in the hospital discharge study. On arrival at the ward, the clinical research nurse was informed that Caleb was rude and agitated and so she should not expect much response or cooperation from him. It was reiterated that he had been admitted due to smoking spice and was a ‘*drug user*’.

On observation, Caleb was sitting on the side of his bed wearing a hospital gown. He had a cut to his forehead and cheek and his face was smeared with dirt. He had cuts to his legs which were bruised and streaked with dirt. He appeared withdrawn and depressed and at first did not seem to want to talk to the research nurse. Caleb was asked what he knew about the plans for him and where his clothes were for when he was discharged. He became animated and distressed as soon as his clothes were mentioned and explained he did not know where they were and that he had lost all his belongings on the street when he was bought in to hospital. As he was not conscious when the ambulance arrived, he was unable to take his bag with him and he assumed that it had been stolen by the people he was with. He was clearly distressed by this and wanted to talk in detail about the belongings and memories he had lost. He explained that he had lost or had stolen most of his belongings while homeless and that this bag had contained his last few possessions and items of clothing. He cried, explaining that he had lost everything and had become homeless after losing his job several months ago. He said he had seen ‘lots of trouble’ on the street but that he had never encountered any before and he was upset and felt shocked by what had happened to him.

His clothes were found by the research nurse in a plastic bag in the bedside cabinet. After 36 h in hospital they were still wet, dirty, smelt of urine and completely unfit to wear. The research nurse was aware these items were potentially the only belongings Caleb owned. She, therefore, washed and dried the clothes on one of the older people care wards that had access to these facilities. Clean clothing, shoes, rucksack, sleeping bag and toiletries were collected from the ‘clothing cupboard’ that had been set up by local government social services for anyone who came into hospital without these items. Caleb was given these items and was extremely happy to shower and change into his new clothes. Caleb was discharged from the hospital later that day and was happy to complete the questionnaire.

### 5.4 Follow up at 3 months (Time 2)

Locating participants to carry out the Time 2 questionnaire was exceptionally challenging. Only 17 questionnaires were completed (as compared to 64 at Time 1). The late appointment of the research nurse to the study was the main barrier as it had left only a 3‐month rather than a 6‐month window for completion of the work. Before leaving hospital, approximately a third of patients gave a mobile telephone number or a forwarding address, usually for a hostel or other form of temporary accommodation. Some gave the address of a specialist homeless GP practice as somewhere their correspondence could be sent. A third of those giving forwarding details were contactable at Time 2 (especially where good relationships had been forged in the hospital). However, following up the remainder was exceptionally time consuming. Most challenging to find were those participants whose stay in hospital was brief or where they were being discharged to the street or had abandoned the accommodation that had been found for them. A good strategy to find people was always to ask about *‘sleep sites’* (i.e. where people might be found if they returned to the street) when collecting initial background information.

Of further importance was developing good relationships with hostel and homeless day centre staff. Initial contact with homeless service providers was made over the phone or email and some were suspicious of the motives for the research. The status of the clinical research nurse as a nurse with a hospital identification badge seemed important in establishing trust with community services. Once suspicion was overcome, the research nurse secured permission from the hostels and day centres to attend in person. The research nurse would arrive early in the morning and join those attending the day centre from breakfast through to lunchtime, after which the day centres closed. This fostered a good working relationship with the staff who then assisted in locating the research participants or informing the research nurse which hostel they had most recently accessed. Consent had previously been obtained from patients to permit this. The research nurse also met *‘by chance’* several participants while attending day centres, walking the streets or at evening street soup kitchens and was able to complete the follow‐up questions opportunistically. This participant observation also provided very useful contextual information for the study.

Once found, most participants appeared happy to see the research nurse and to take part in the follow up. Again, this would often lead to a lengthy encounter as participants were keen to talk in detail about what had happened to them on the streets since their discharge from hospital. Other participants were difficult to interview owing to substance misuse and had to be approached at unsocial times of the day. One participant who was *‘street drinking’* could only answer a couple of the questions at a time as they became too emotional and tearful while being asked. If the research nurse did not locate them on the streets by 9:00 AM, they would be too intoxicated to take part.

Where possible, the research nurse attended homeless services, sleep sites or the person's own home with another member of the research team or otherwise left details of location and timings, reporting back once the visits were safely concluded. Visits mostly went well, but, on a small number of occasions, the research nurse felt the potential for threat. It is important that research nurses have some awareness of psychologically informed practices such as those described by Schout et al. (2009) for managing the potential for conflict as well as taking all practical measures such as carrying personal alarms.

### 5.5 ‘Plugging the gap’

It was often the case that once the research nurse found a study participant, it was not a straightforward encounter that involved running through the questionnaire. Participants were often experiencing some form of crises or emotional upset and this could involve considerable amounts of time alerting services or providing some comfort and time to talk through matters. The fact that the research nurse had nursing skills was invaluable. On a number of occasions, the research nurse had to persuade participants that they needed to see a GP urgently or go to the emergency department (ED). Often the research nurse would need to accompany them and help advocate for them on arrival. There were regular late evenings spent in ED sitting with patients who would otherwise leave if left alone, usually due to the onset of unpleasant withdrawal symptoms associated with drug or alcohol use disorder. The research nurse also regularly encountered HIV+ participants who were not taking their antiretroviral medication and missing essential hospital appointments either due to being too depressed to attend or too high or intoxicated to remember. Thus, follow‐up visits that should have taken around an hour for questionnaire completion would end up lasting several hours because of the health needs and social circumstances of the participant.

Hostel staff were also often relieved to see a nurse on their doorstep and would ask for advice about how to manage the health needs of their residents. Some hostels did not have access to a specialist homeless community nurse and were trying to manage complex health conditions by themselves. Shortages of hostel staff meant they could not accompany residents to healthcare services.

The research nurse role during this study often felt as though it was *‘plugging a gap’* in community health and care services and enabling both basic medical and pastoral care, including referrals to other primary and secondary services, to be carried out alongside the project. Over time this impacted on the research nurse who started to experience feelings of burnout and was regularly finding her day shifts lasting 13 h. As it was not always possible to do everything that was needed for a patient, this led to feelings of guilt and frustration and a sense of having never done enough. The work was often described as traumatising, seeing, for example, the impact of practices such as discharge to the street where patients with whom the research nurse had formed close bonds were to be found sometimes unconscious lying on the pavement or returning to hospital badly assaulted. Several safeguarding referrals to social care services were made by the research nurse and the research team, but these often did not provide the immediate help that was needed (e.g. somewhere safe to stay).

### 5.6 Performance management targets

As a result of the time it took to follow up homeless patients in the community, the research nurse came under pressure from her manager at the hospital who expressed concern that the homeless study was taking up too much of her time. In addition to the homeless study, there was pressure to meet recruitment targets for the clinical trials for which she was also responsible. There seemed little understanding of why a homeless study, with such a seemingly low recruitment target, should take up so much time. Current performance management frameworks targets do not usually have adjustments for different types of participants (e.g. those described as *‘hard to reach’*) or study designs that require follow up of the same participant more than once and in such diverse settings. When stepping outside of NHS settings (the usual workplace), time is also needed to build relationships with service providers who will be instrumental later on in supporting the research nurse's ability to deliver the study. This is especially likely in respect of the diverse settings and range of providers that characterise community, social care and public health. Finally, given the often emotional and distressing nature of this research work, it was felt that the existing supervision arrangements needed enhancing and future research nurses might benefit from being able to access the psychological informed supervision that is offered to others working in the homeless sector. The complex trauma experienced by many who are homeless can provide additional challenges for research nurses in forming and managing the relationships we have argued are important for recruitment and sustaining involvement in research.

## 6 DISCUSSION

This case study demonstrates ways in which clinical research nurses can make an important and unique contribution to social care research. However, the challenges presented by methods that differ from clinical trials, within more diverse contexts and with complex client groups, require an expanded skill set. In clinical trials, research nurses sometimes feel under considerable pressure to meet recruitment targets (Tinkler et al., 2017). This can lead to low job satisfaction and poorer performance in circumstances where the research nurse suspects that the study is unlikely to benefit the patient or has not been properly explained to them. In this study, the *caring*‐*recruitment* dichotomy was differently framed, reflecting the need for different performance management, training and support requirements. Here, study participants were often experiencing the most extreme forms of social exclusion. This posed significant logistical challenges (such as finding *‘sleep sites’*) and also required specialist clinical knowledge to understand why, for example, a patient may be agitated (e.g. because of drug withdrawal symptoms) and findings ways to manage this so that the participant could still take part in the study (e.g. visiting at the optimal time of day). Inadequate protections and shortages of community‐based care also posed problems leading to a blurring of boundaries between the role of the research nurse and the complex case manager. Here, recruitment to the study often depended on the clinical research nurse taking on some form of temporary responsibility for unmet care and support needs. Other studies have reported similar experiences when working with patients who are homeless. Brown et al. (2020), for example, report how in a US study of homelessness the researchers often found themselves having to advocate for study participants themselves as overloaded homeless outreach were at full capacity. This *‘plugging the gap’* can lead to stress and possible burnout, especially where there is a lack of system support with regard to the impact this can have on their ability to meet wider recruitment targets for the other studies they may be involved with. There is a risk that this caring role may lead to neglect of other studies on the portfolio because of the perceived benefits to homeless patients. In some instances, there was a great sense of personal accomplishment in thinking that participants may not have accessed the emergency services they needed had the research nurse not tracked them down. Entering the field of homeless service provision was also something of a culture shock for the research nurse having been previously hospital based. Having the back‐up and support of the wider research team where there was expertise in homelessness was especially important. Just prior to the outbreak of the Coronavirus pandemic, the CRN had planned to host a series of seminars looking at issues such as how to recruit patients who were *‘hard to reach’*. Expanding training and learning networks for clinical research nurses in these new areas will be particularly key to underpinning the success of this specialist workforce development.

## 7 LIMITATIONS

The limitations of this present study are that it draws on one subjective example where the involvement of clinical research nurses was opportunistic rather than well planned from the outset. Its strengths lie in its close‐to‐practice perspective and the analysis being situated in the wider literature. This has enabled important learning for: the NIHR and the CRN on the workforce issues that can underpin the successful delivery of social care research; and for academics and social care researchers developing studies through making the value of research nurses more visible.

## 8 CONCLUSION

Clinical research nurses can be a significant asset in extending support to social care research, especially in terms of supporting the participation of those whose voices are rarely heard. However, if this is to be most effective, the systems around research nurses need to be flexible to adapt to the demands of these new study contexts. Reflections on the *‘care*‐*recruiting’* dichotomy indicate how different types of research may require different types of emotional labour, creating different ethical challenges and placing different pressures on research nurses. It is important to be mindful that maintaining the equilibrium between research and care delivery in the clinical research nurse role can become increasingly fraught where there are service shortfalls. Facilitating learning networks and good supervision will, therefore, be key to retaining clinical research nurses in these new and expanded roles.

## 9 RELEVANCE FOR WORKFORCE DEVELOPMENT

This study highlights some of the challenges facing clinical research nurses in working in social care and homelessness research where participants may be identified as *‘hard to reach’*. If research nurses are to work effectively in these settings and to avoid burnout, then it is important to ensure that performance management, training and supervisory arrangements are adapted accordingly.

## CONFLICT OF INTEREST

No conflict of interest has been declared by the authors.

## AUTHOR CONTRIBUTIONS

All authors have confirmed that all authors meet the ICMJE criteria for authorship credit (http://www.icmje.org/recommendations/browse/roles‐and‐responsibilities/defining‐the‐role‐of‐authors‐and‐contributors.html) as follows: Substantial contributions to the conception or design of the work; or the acquisition, analysis or interpretation of data for the work; Drafting the work or revising it critically for important intellectual content; Final approval of the version to be published and Agreement to be accountable for all aspects of the work in ensuring that questions related to the accuracy or integrity of any part of the work are appropriately investigated and resolved.

## ETHICAL APPROVAL

Ethics approval for the study was secured from the National Research Ethics Committee [REC Ref: 16/EE/0018].

## Data Availability

The data that support the findings of this study are available from the corresponding author upon reasonable request.
